# Brood Reduction via Intra-clutch Variation in Testosterone - An Experimental Test in the Great Tit

**DOI:** 10.1371/journal.pone.0056672

**Published:** 2013-02-20

**Authors:** Katarzyna Podlas, Fabrice Helfenstein, Heinz Richner

**Affiliations:** 1 Institute of Ecology and Evolution, University of Bern, Bern, Switzerland; 2 Institute of Biology, University of Neuchâtel, Neuchâtel, Switzerland; CNRS, Université de Bourgogne, France

## Abstract

In birds, yolk androgen concentrations in eggs can increase or decrease over the laying sequence and common hypotheses hold that this serves to favour the competitive ability of either first- or last-hatched chicks depending on the prevailing conditions, and thus promote brood reduction or maintenance of original brood size respectively. Intra-clutch variation of testosterone can shift relative competitive ability of siblings and hence competitive dynamics. In a natural population of great tits, we experimentally investigated the effects and function of maternal testosterone on offspring phenotype in relation to the laying position of the egg in a context of hatching asynchrony. To this end, we created three types of clutches where either the first three or the last three eggs of a clutch were injected with testosterone (T) dissolved in sesame oil, and the remaining eggs with sesame oil only, or where all eggs of a clutch were injected with sesame oil. Increased levels of yolk T in the last-laid eggs resulted in the last-hatched chicks being significantly lighter and smaller than their siblings, while increased levels of T in the first-laid eggs had no direct effect on the first-hatched chicks, but an indirect negative effect on their siblings. Our results suggest that females can potentially adjust offspring phenotype by modulating, over the laying sequence, the amounts of T deposited in the eggs. These results are in contradiction, however, with current hypotheses and previous findings, which suggest that under good conditions higher levels of maternally derived T in the last-laid eggs should mitigate the negative effects of hatching asynchrony.

## Introduction

Maternal effects are defined as modifications of offspring phenotype caused by the maternal phenotype or the environment that mothers experience [1]. Maternal effects are a prime mechanism in the control of phenotypic variation [1] and have been demonstrated across a wide variety of living organisms in both plants e.g. [2]–[4] and animals e.g. [5], [6]. Of the many potential mediators of maternal effects, maternally-derived hormones [7]–[10] are especially interesting since hormones are well-known to play a major role in organizing phenotypic differentiation and regulating physiological functions [11].

Among maternally-derived hormones, testosterone (T) has received particular attention [12]–[14] since it can have profound effects on embryo development [7], [15]–[18] with short- and long-term consequences for both offspring and adult behaviour [19]–[25]. High concentrations of maternally-derived T may increase post-natal growth [17], [26]–[29] and offspring competitiveness in sibling interactions [16], [28], [30]–[33]. Its anabolic properties [34] may lead, for instance, to accelerated growth of the neck muscles involved in begging or sucking [35]–[37]. Besides these beneficial effects, high levels of maternal T have also been shown to have costs [7], [38]–[40]. Yolk T may directly suppress immune functions [41–44, but see 45], contribute to metabolic dysfunctions (e.g. hyperinsulinemia, [46]), increase oxidative stress [47], or have indirect costs via trade-offs in resource allocation caused by accelerated growth (e.g. reproductive anomalies at adulthood [17], impaired cognitive ability [48], reduced life span [49]). Whether deposition of high levels of T in the yolk comes at a cost for the females is still debated. Some studies found that transferring high amounts of T to the embryo or into the eggs may inflict costs to the mother (reviewed by [21], [22]), while others provide evidence that the deposition of high levels of T in the yolk does not require elevated levels of circulating androgens, thus suggesting no cost to the female [9], [50]. Nevertheless, owing to the potentially opposing costs and benefits of maternally-derived T for offspring, and to some extent for females, mothers are expected to optimize the allocation of testosterone to the embryo or egg (reviewed in [9], [21]). Strategic allocation of T would then underlie variation in the levels of T found in the egg yolk or in the blood of neonates across clutches or litters produced by different mothers [16], [51]. Furthermore, it may also underlie variation in the levels of T found in the egg yolk or in the blood of neonates within the clutch or the litter of a given mother [7], [52], [53]. This latter pattern of variation in the levels of maternally-derived T across eggs or neonates is mostly seen in oviparous and ovoviviparous species where mechanisms allowing the mother to adjust the amount of T deposited into each of the eggs are more likely to have evolved [7], [14], [21], [28].

In birds, the concentrations of T deposited in eggs may increase [14], [54] or decrease [55], [56] with laying order, depending on the taxon. The within-clutch variation in maternally-derived T described in birds [14], [57] has been interpreted as an adaptive tool to adjust brood size to food availability [9], [58]. Hatching asynchrony occurs in a wide variety of bird species. It is supposed to result from an onset of incubation before full clutch completion [59]. Asynchronous hatching establishes an age hierarchy within a brood, which may then result in a competitive advantage for older, first-hatched chicks over their younger siblings [60]. The combination of hatching asynchrony and varying levels of yolk T can influence offspring growth and begging ability, and may thus allow mothers to adaptively tune the survival prospects of the offspring to the conditions prevailing during egg laying and anticipated conditions during brood rearing [61]. It has been hypothesized that by depositing higher levels of T with increasing laying order, females could mitigate the negative consequences of sibling asymmetry by increasing the competitive ability of the chicks hatched from the last-laid eggs when food is plentiful, or handicap those chicks when food is scarce [7], [14], [28], [58], [62]. Higher T-levels induce metabolic costs (e.g. oxidative stress [58]) also for chicks. These costs may be more than compensated by the benefits of T-induced higher competitiveness under good food conditions, but become a handicap under bad conditions [7], [58].

The opposite pattern, i.e. allocation of higher levels of T in first-laid eggs, could exacerbate disadvantages for later hatchlings, facilitating brood reduction under bad food conditions [63]. In this latter case, the higher levels of maternally-derived T in the first-laid eggs would boost the competitive ability of the first-hatched chicks and precipitate brood reduction via the death of the less competitive chick(s) hatched from the last-laid egg(s) [9], [56].

The fact that strong natural variation in T concentration occurs *within* clutches is of importance since it potentially affects the dynamics of family interaction and sibling competition by creating variation in competitive ability. It may thus lead to a different outcome than the more frequently studied variation in T concentrations among clutches where all siblings are potentially rendered more or less competitive. To our knowledge, only a few studies manipulated the levels of yolk T within clutches directly, and investigated the adaptive significance of varying concentrations of yolk T in relation to the laying order in a context of hatching asynchrony. Examples include Black-headed gulls [31], [62], Canaries [64], American kestrels [65], and Zebra finches [66]. Although it is generally assumed that within-clutch variation in maternal T serves to mitigate the negative effects of hatching asynchrony on last-hatched offspring, contradictory findings mainly due to differences in methods used, call for additional work to better understand the functional significance of within-clutch variation in T concentration. Most of the previous studies tested the effects of T by experimental creation of clutches were T in first-laid eggs was increased to the levels of the last-laid eggs. However the manipulated clutches contained eggs originating from different mothers, with the potential problems of an inflation of within-brood variance in offspring traits through genetic effects and gene-by-environment effects, a modification of the clutch sex-ratio and a disruption of within-clutch T-allocation patterns. To our knowledge, only one study compared the effects of high yolk T in a context of hatching asynchrony by manipulating levels of T in both first and last-laid eggs at the same time [64].

We conducted a field experiment where we manipulated within-clutch T concentration in great tit *(Parus major)* eggs in order to assess whether higher levels of yolk T enhance the growth and survival of the first- or last-hatched chicks and thus would be compatible with a brood reduction or a compensatory strategy respectively. We created three types of clutches: (1) clutches where the first three eggs were injected with T and the remaining eggs with sesame oil, (2) clutches where the last three eggs were injected with T and the remaining eggs with sesame oil, and (3) control clutches where all eggs were injected with sesame oil. We thus manipulated yolk hormone concentrations of eggs to mimic the natural variation in yolk T with laying order within clutches. The experimental design and predictions are based on the findings (e.g. [63], [67]) that, although the general pattern in great tits shows higher levels of T in the last-laid eggs, some females still lay clutches where the reversed pattern is observed, i.e. first-laid eggs contain more testosterone (shown for great tits by [63], [67]). We thus created three experimental groups aiming to: 1) test the effects of T on chicks in relation to the laying position of the eggs from which those chicks hatched (within-brood comparison), 2) test the effects of T on chicks by comparing the chicks from first- or last-laid eggs with experimentally elevated T levels with chicks from first- or last-laid oil-injected eggs in control broods (between-brood comparison), and 3) test whether testosterone has positive or negative effects on chicks whatever their rank (positive internal control = broods where testosterone was injected into the first-laid eggs).

In this study hatching success was reduced following injection (see below), and thus inadvertently created an experimental situation where food conditions were improved and sibling competition weakened. Reduced brood size is well known to have strong positive effects on chick quality, fledging success and parental condition in great tits [68]. Consequently, we decided to narrow our predictions down to the situation of good food conditions. Therefore, we predicted that in broods where T is injected into the first-laid eggs, first-hatched chicks should achieve higher fitness (grow larger and heavier and have higher fledging success) than last-hatched chicks within the same brood. Compared to first-hatched chicks in control broods, T-injected first-hatched chicks may perform equally well or better depending on how strongly additional yolk T will alter their ability to monopolize food and parental care. Last-hatched chicks in broods where T is injected in first-laid eggs are predicted to perform equally or less well than last-hatched chicks in control broods, again depending on how strongly the T-injected first-hatched chicks will be affected by the additional yolk T. In contrast, we predicted that in broods where T is injected into the last-laid eggs, the last-hatched T-injected chicks should do equally well (full compensation) or slightly worse (partial compensation) than first-hatched chicks within the same brood, but should in any case do better than last-hatched chicks in control broods. In control broods, we expect hatching asynchrony to induce last-hatched chicks to grow smaller and lighter than their first-hatched siblings.

## Materials and Methods

### Study Site and Model Species

The experiment was performed in spring 2009 in a wild population of great tits, breeding in nest-boxes in the Köniz forest near Bern, Switzerland (46°56′ N, 7°24′ E). The study was approved by the Ethical Committee of the Agricultural Office of the Canton Bern, Switzerland (experimentation permit 25/09) and the Federal Agency for Environment of the Canton Bern, Switzerland (ringing permit 2819). Hatching asynchrony with hatching spread up to 3 days is common in this species e.g. [69] and last-hatched chicks frequently die before fledging. From early March, we visited nest-boxes regularly to determine the start of nest building and egg-laying. After the laying of the first egg we visited nests every day and numbered eggs consecutively.

### Experimental Procedure

After clutch completion, three types of experimental clutches were created where either (1) the first three laid eggs were injected with T (thereafter called “T-first” clutches) and the others with 5 µl of sesame oil, or (2) the last three eggs were injected with T (“T-last” clutches) and the others with 5 µl of sesame oil, or (3) all eggs were injected with 5 µl of sesame oil (“control clutches”).

For T injections we used 15 ng of T (17β-hydroxy-4-androsten-3-on, Fluka, Switzerland) dissolved in 5 µl of sesame oil. Eggs were injected after clutch completion to limit the disturbance to females during egg laying and to keep our workload within feasible daily limits. To ensure correct statistical analyses and mimic higher levels of T in either the first- or last-laid eggs, we have chosen to inject more than one egg. The amount of T injected in this study fits into the range of concentrations of yolk T previously found in the same species (e.g. females in captivity: concentrations are given, which calculate as approx. mean: 5.5 ng/yolk assuming an average yolk weight of 0.34 g as found in several studies [63]; females in natural population: mean: 8.87 ng/yolk, range: 1.7–30 ng/yolk [67]; females in natural population: concentrations are given, which calculate as approx. mean: 19 ng/yolk [70]). Also, a precursor of testosterone, androstenedione A4, is found in considerably higher quantities than T in all three studies mentioned (e.g. concentrations are given, which calculate as approx. mean: 12.5 ng/yolk assuming average yolk weight of 0.34 g as found in several studies [63]; mean: 18.5 ng/yolk, range: 2.7–60.8 ng/yolk [67]; concentrations are given, which calculate as approx. mean: 16.3 ng/yolk [70]). Since A4 can be converted to T by the embryo even at early stages [9], [71], the injected dose of T may thus be in a physiological range that is relevant for addressing its functions in brood reduction. The maximal amount of T found in great tit eggs of a neighbouring population was 30 ng/yolk [45], [67]. A previous study injected this exact amount into egg yolks and found positive effects on chick growth [45].

All injections were performed in the field. During manipulation, the eggs were replaced with dummy eggs in the nest for approximately a half hour. Before injection we cleaned a small spot on the egg surface with 70% ethanol. We used a 25 µl syringe (Hamilton 702LT) with a 25-G needle for injections and a cold light source to monitor whether the needle entered the yolk membrane. After the injection the hole in the eggshell was sealed with a small drop of a tissue adhesive (Nexaband S/C Topical Tissue Adhesive, England; for method see [45]).

In order to assign each chick to its egg of origin, and thus the treatment it experienced, we injected all the eggs with 2 µl of alimentary, non-toxic colorant one day before the predicted hatching date. Within a clutch, the eggs of the different treatments (T or sesame oil) received different colours. The colorant sticks to the hatchling and is visible after hatching and thus allows individual identification. The colour used with respect to treatment and position in the laying sequence was assigned randomly (red or green).

A total of 406 eggs were injected, out of which 224 chicks hatched. The overall hatching success, as measured by the number of hatched chicks over clutch size, was 55.2% and it did not significantly differ among the experimental treatments (*χ^2^_2_* = 1.27, *P = *0.53, n = 51).

### Nest Monitoring

Around the predicted date of hatching, we visited the nests every day to determine the hatching date of the first chicks in the brood (day 0). In our study population hatching spread was 0.72+/−0.70 day (71% chicks hatched on day 0, 26.8% hatched on day 1 and only 2.2% hatched on day 2). On day 3, when all chicks had hatched, we individually marked chicks by removing specific combinations of tuft feathers, and could assign them unequivocally to the previous treatment since the colorants were still clearly visible. Chicks were ringed 8 days after hatching with an aluminium ring (Vogelwarte, Sempach, Switzerland). Thirteen days after hatching chicks were weighed to the nearest 0.1 g and their tarsus and wing lengths were measured to the nearest 0.1 mm. Nests were checked daily from day 16 onwards to record the number of young fledged and the fledging date.

### Assessment of Yolk T concentrations

In 2010, on the day of laying, each egg of 17 clutches was numbered according to its laying order, removed from the nest and replaced by a dummy egg until clutch completion. In total, we collected 134 eggs (from 4 to 11 eggs per clutch) and stored them at −80°C until analyses of yolk T concentrations.

Yolk T concentrations were assessed by enzyme immunoassay (EIA) following the protocol described in Palme and Möstl [72] and Möstl et al. [73]. To extract the yolks we scraped the egg-shell and albumen from the frozen eggs using a scalpel. Yolks were weighed and a fraction of each (0.15 g) was homogenised in 600 µl of distilled water, vortexed for 30 s and frozen. On the next day, samples were thawed and 3 ml of 100% methanol added. They were then shaken for 30 min and again frozen overnight. The next day, samples were thawed, centrifuged for 15 min at 2500 G, and 1 ml of the supernatant transferred into a new vial. Methanol was evaporated under a stream of nitrogen, and the solid residues were re-suspended in 500 µl of assay buffer (for details see [72] and [73]). In duplicates, 10 µl were directly measured in a testosterone enzyme immunoassay. Intra- and inter-assay coefficients of variation were 9% und 16%, respectively. Sensitivity of the assay was 0.99 pg/well. Minimum detectable hormone level was 1.24 ng immunoreactive T per g of yolk.

### Statistical Analyses

Analyses were done using the R software (R Development Core Team 2007, [74]). To test whether the amount of yolk T varied according to the laying order we used linear mixed models with normal distribution of errors. To overcome the potential bias that would be generated by a positive covariation between clutch size and overall T levels in the eggs (i.e. eggs from large clutches containing more T), we centred yolk T concentrations by subtracting the clutch mean from each value of yolk T [75]. Laying order of eggs (ranging from 4 to 11) was included in the model as a covariate and brood identity as a random factor. Additionally, we tested whether the average amount of T was related to clutch size using a general linear model with normal distribution of the error.

To test the randomisation of our treatments for clutch size, we used a generalized linear model with Poisson errors where treatment was included as a fixed factor. A general linear model with normal distribution of errors was used to investigate whether brood size on day 3 differed according to the treatments.

To test whether laying order of eggs predicts hatching order of the chicks we used generalized mixed models with binomial errors. Hatching spread was limited in our study with only 2.2% of the eggs hatching 2 days after the first ones had hatched. We therefore pooled the later hatched chicks to create a binary response variable: hatched first vs. hatched later ( = hatched 1 and 2 days later). Fixed factors and covariates were as follows: position of the egg in the laying sequence (two levels factor: first or last), clutch size and laying date of the first egg in the clutch. The brood identity was entered as a random factor.

To investigate the effects of elevated levels of yolk T in first- and last-laid eggs on chick morphology we conducted two types of comparisons. First, we did within-brood comparisons where we tested the effects of T on chicks in relation to the laying position of the eggs from which those chicks hatched. Second, we conducted all pair-wise comparisons between broods to test our predictions (see Introduction) and to investigate the performance of each type of chick relative to all other chicks from control and T-injected broods. In particular, comparing chicks hatched from first- or last-laid T-injected eggs to chicks of control broods (oil-injected) hatched from eggs with the same position in the laying sequence tests the prediction that T-injected chicks should perform better or equally well as control chicks. Also, a competitive disadvantage for last-hatched chicks over their T-injected, first-hatched siblings would be evidenced through last-hatched, oil-injected chicks being smaller than last-hatched chicks from control broods. A neutral effect on last-hatched chicks of having T-injected siblings would be evidenced through oil-injected chicks performing as well as last-hatched chicks from control broods.

Morphological traits were analysed separately using general linear mixed models (nlme package, [76]), thus allowing to estimate treatment effects on each trait and possible trade-offs between traits. Individual fledging probability was modeled using generalized linear mixed models with binomial errors (binary response variable: fledged vs. non-fledged; MCMCglmm package, [77]). For within-brood comparisons, models included laying position, treatment and their two-way interaction as fixed factors, and brood identity as a random factor. For between-brood comparisons, models were run separately using only chicks involved in specific comparison (e.g. first-hatched T-injected from T-first broods and first-hatched from control broods). The latter models included the position of the egg in the laying sequence as a fixed factor, and brood identity as a random factor. All models additionally included brood size on day 3 and laying date as covariates.

For a comparison of the length of the brood rearing period among treatment groups, we used a general linear model where the treatment was included as a fixed factor, and the clutch size and the laying date as covariates. Whole-brood fledging success, computed as the number of fledged young over the brood size at hatching, was analysed using a generalized linear model with binomial distribution of the error, where the treatment was included as a fixed factor, and the clutch size and the laying date as covariates.

Model residuals were inspected to identify deviations from assumptions of normality, linearity and homoscedasticity. Non-significant interactions (*P*>0.10) were stepwise backward eliminated starting with those of highest P-values. Fixed effects were tested for significance using two-tailed, type II F-or χ^2^-tests, except for generalized mixed models using a binomial distribution of the error, for which we used Monte Carlo Markov Chains (MCMC) simulations using the R package MCMCglmm [77]. These models provided the parameter estimates of the fixed effects along with their 95% confidence intervals and associated p-values. Significance of the interaction in generalized mixed models with binomial structure of errors were analysed using *anova* command which compares the two models (with and without interaction) using an analysis of deviance [78]. In one brood the information on body mass was missing.

## Results

### Hormone Levels

**Figure 1:**
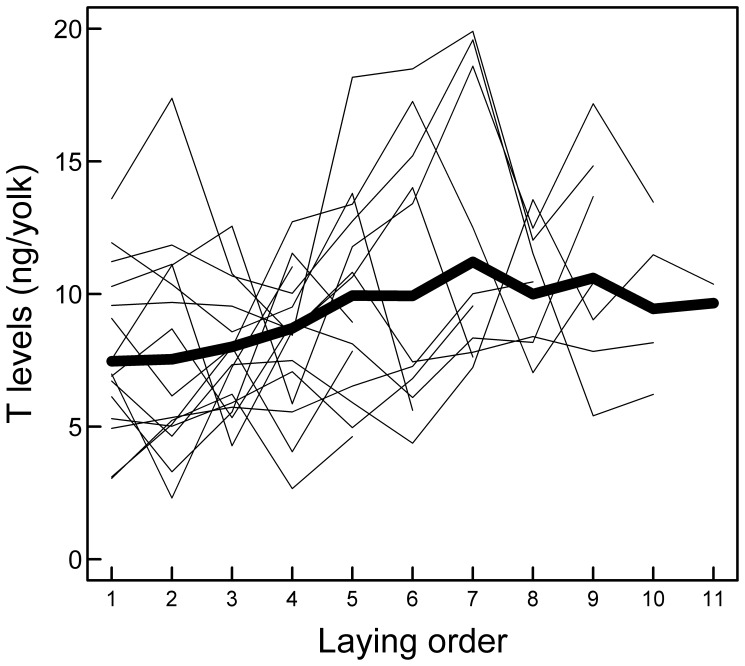
Within-clutch variation in yolk T (ng/yolk). Black thin lines indicate levels of yolk T according to the laying order for each female. The black bold line indicates mean yolk T levels according to the laying order.

Data from 2010 show that the yolk T content significantly increased with laying order (within clutch regression using clutch-centered yolk concentrations: *F*_1,117_ = 10.29, *P* = 0.002, mean: 8.25±2.83 ng/yolk, range: 2.31–20.49 ng/yolk; concentration: 28.79±9.25 ng/g of yolk, Figure 1) supporting results from previous studies on the same species [67]. Average T levels per clutch was not significantly related to the clutch size (*F*_1,15_ = 3.40, *P* = 0.085).

### Validation of Experimental Design

A total of 51 great tit clutches were manipulated (15 control clutches, 18 T-First clutches and 18 T-Last clutches). Clutch size was randomized over treatments (*χ^2^*_2,50_ = 1.16, *P* = 0.56, mean clutch size ±1 SE: 8.60±0.37 egg for control clutches, 7.50±0.32 egg for T-First clutches, 7.83±0.33 egg for T-Last clutches). Brood size on day 3 did not differ among treatments (*χ^2^*_2,50_ = 3.18, *P* = 0.20, mean brood size on day 3±1 SE: 5.40±0.50 chick for control clutches, 4.80±0.39 chick for T-First clutches, 4.00±0.34 chick for T-Last clutches).

First, verifying that the position of the eggs in the laying sequence predicted their hatching order, we found that last-laid eggs were significantly more likely to hatch at least 1 day later than the first-laid eggs (position of the egg in laying sequence: estimate [95% CI] = 0.14 [0.02; 0.25], *P* = 0.02, brood size on day 3: estimate [95% CI] = −0.03 [−0.08; 0.01], *P* = 0.20, laying date: estimate [95% CI] = 0.008 [0.001; 0.02], *P* = 0.06).

### Within-brood Comparisons

**Figure 2:**
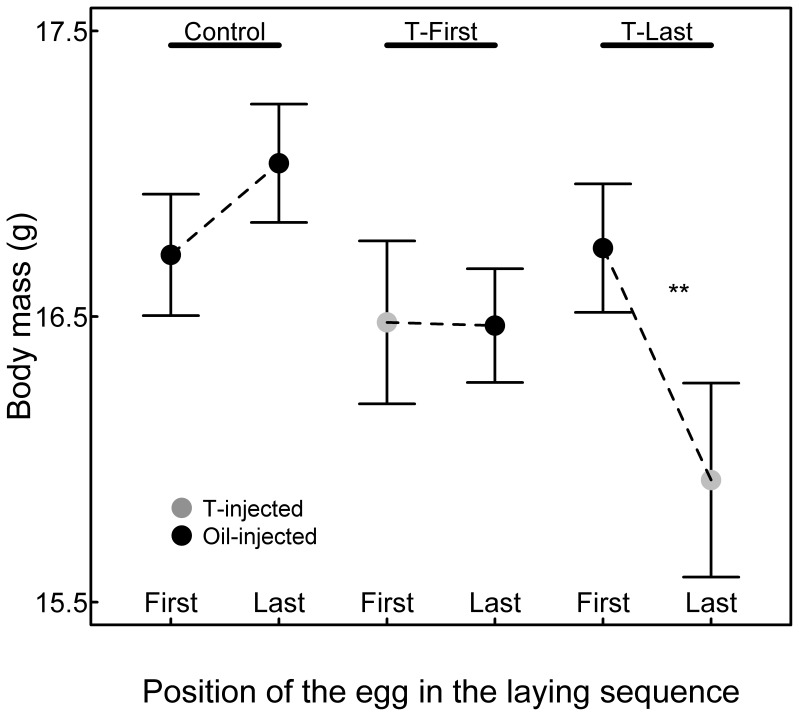
Body mass (mean ± SE) of chicks in relation to the laying position and treatments. Grey points indicate body mass of chicks hatched from T-injected eggs. Black points indicate body mass of chicks hatched from oil-injected eggs.

**Figure 3:**
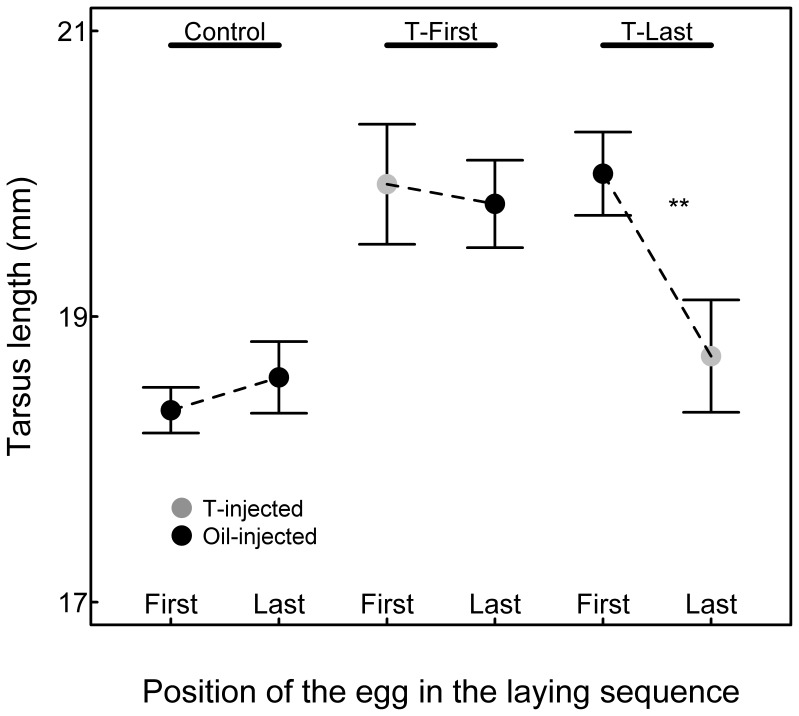
Tarsus length (mean ± SE) of chicks in relation to the laying position and treatments. Grey points indicate chicks hatched from T-injected eggs. Black points indicate chicks hatched from oil-injected eggs.

**Figure 4:**
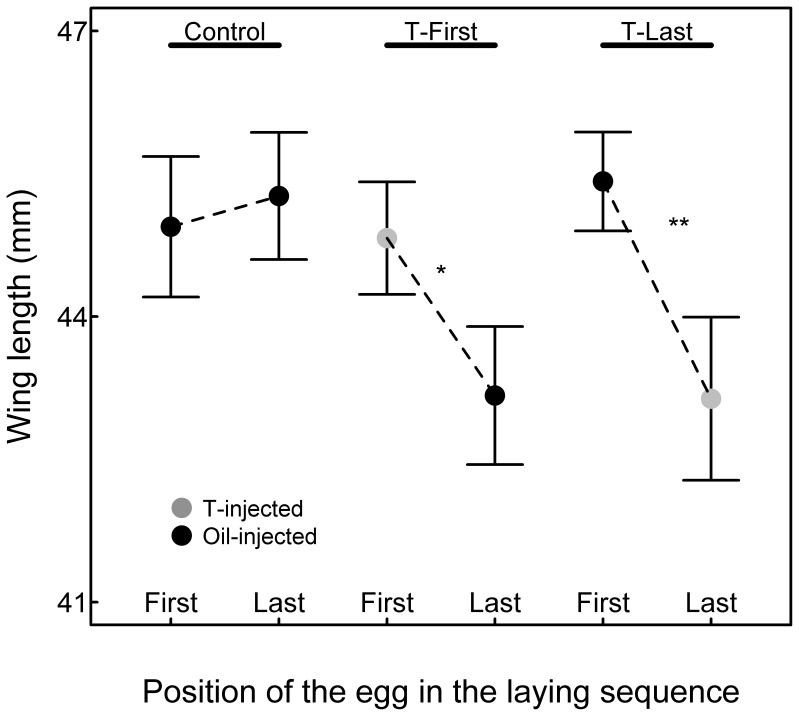
Wing length (mean ± SE) of chicks in relation to the laying position and treatments. Grey points indicate chicks hatched from T-injected eggs. Black points indicate chicks hatched from oil-injected eggs.

Size and body mass of 13 days old chicks were differently affected depending on the laying position in the three experimental groups (significant interaction between laying position and treatment; Table 1; Figures 2, 3 and 4). In control broods, chicks hatched from first-laid eggs had similar body mass and size as their siblings hatched from last-laid eggs (mean difference ±1SE: body mass: 0.39±0.22 g, post-hoc t-test: t_1,159_ = 1.78, P = 0.076; tarsus length: 0.13±0.19 mm, post-hoc t-test: t_1,160_ = 0.71, P = 0.48; wing length: 1.01±0.75 mm, post-hoc t-test: t_1,160_ = 1.35, P = 0.18). In T-Last broods, chicks hatched from first-hatched oil-injected eggs were heavier and bigger than their siblings hatched from last-laid T-injected eggs (mean difference ±1SE: body mass: 0.78±0.24 g, post-hoc t-test: t_1,159_ = 3.26, P = 0.002; tarsus length: 0.92±0.21 mm, post-hoc t-test: t_1,160_ = 4.44, P<0.001; wing length: 1.97±0.81 mm, post-hoc t-test: t_1,160_ = 2.43, P = 0.016). In T-First broods, chicks hatched from first-laid T-injected eggs had similar weight and tarsus length but longer wings than their siblings from the last-laid oil-injected eggs (mean difference ±1SE: body mass: 0.26±0.22 g, post-hoc t-test: t_1,159_ = 1.20, P = 0.23; tarsus length: 0.17±0.19 mm, post-hoc t-test: t_1,160_ = 0.89, P = 0.37; wing length: 1.98±0.73 mm, post-hoc t-test: t_1,160_ = 2.70, P = 0.008).

**Table 1:** Within-brood analysis of the effects of an injection of T and the laying position on chick size and mass.

	Body mass			Tarsus length			Wing length		
	*F*	df	*P*	*F*	df	*P*	*F*	df	*P*
Laying position	1.43	1,159	0.23	0.50	1,160	0.48	1.82	1,1600	0.18
Treatment	0.16	2,46	0.85	**4.54**	**2,45**	**0.016**	0.93	2,45	0.40
Brood size	0.12	1,44	0.73	0.02	1,44	0.89	**6.61**	**1,45**	**0.013**
Laying date	1.64	1,45	0.21	3.32	1,45	0.075	1.03	1,44	0.32
Laying position x treatment	**6.56**	**2,159**	**0.002**	**7.31**	**2,160**	**<0.001**	**5.20**	**2,160**	**0.006**

Significant effects are highlighted in bold.

Fledging probability of individual chicks did not differ among experimental groups and was not affected by laying position (laying position: estimate: 0.005, CI = [−0.03; 0.04], *P* = 0.76; T-First broods: estimate: 0.04, CI = [−0.09; 0.19], *P* = 0.65; T-Last broods: estimate: 0.09, CI = [−0.07; 0.24], *P* = 0.22; laying position x treatment: *χ^2^*_2,160_ = 3.21, *P* = 0.21; brood size: estimate: 0.005, CI = [−0.03; 0.04], P = 0.82; laying date: estimate: 0.003, CI = [−0.009; 0.003], P = 0.36).

### Between-brood Comparisons

Between-brood comparisons of body mass and wing length all yielded non-significant results (analyses for body mass: all *F* <3.56, all *P*>0.07; analyses for wing length: all *F* <3.61, all *P*>0.086).

All chicks from control broods (oil-injected eggs) grew shorter tarsi than chicks hatched from both types of eggs in T-First broods (mean difference ±1SE with first-laid T-injected: vs. last-laid control oil: 1.56±0.73 mm, *F*_1,30_ = 4.56, *P* = 0.041; vs. first-laid control oil: 1.53±0.70 mm, *F*_1,30_ = 4.85, *P* = 0.035; mean difference with oil-injected ±1SE: vs. last-laid control oil: 1.36 mm ±0.62, *F*_1,30_ = 4.79, *P* = 0.037; for first-laid control oil: 1.33±0.59 mm, *F*_1,29_ = 5.08, *P* = 0.032) and than chicks from first, oil-injected eggs in T-Last broods (mean difference ±1SE: vs. last-laid control oil : 1.50±0.58 mm, *F*_1,28_ = 6.64, *P* = 0.015, vs. first-laid control oil: 1.51 mm ±0.53, *F*_1,28_ = 8.19, *P* = 0.008). However, they grew tarsi of similar size to chicks hatched from last-laid, T-injected eggs (*F* <0.83, *P*>0.37). No difference in tarsus length however was observed between chicks form T-First and T-Last broods (all *F* <2.11, all *P*>0.15).

All types of chicks achieved similar individual fledging probability (all estimates <0.12, *P*>0.14).

### Brood Rearing Period and Whole-brood Fledging Success

We found no significant effect of the treatment on the length of the brood rearing period (mean value ±1SE for control broods: 18.78±0.40 days; mean value ±1SE for T-First broods: 19.41±0.40 days; mean value ±1SE for T-Last broods: 19.05±0.39 days; Treatment: *F*_1,45_ = 0.85, *P = *0.43, clutch size: *F*_1,44_ = 0.001, *P = *0.99, laying date: *F*_1,47_ = 3.46, *P = *0.07). Finally, there was no difference in whole-brood fledging success among experimental broods (mean fledging success for control broods: 90%; mean fledging success for T-First broods: 85%; mean fledging success for T-Last broods: 92%; *χ^2^_2_* = 0.14, *P = *0.93).

## Discussion

The aim of this study was to investigate the effects of maternal testosterone on offspring phenotype in relation to the laying position of the egg, and its function in a context of brood size adjustment. Overall, we found that experimentally elevated levels of T affected chick growth differentially depending on the laying position. Increased levels of T in later laid eggs had a negative effect on chick growth, while increased levels of T in the first-laid eggs had no effect on the first-hatched chicks, but an indirect negative effect on their last-hatched siblings. Our results do not support the “compensation” strategy hypothesis, i.e. the hypothesis that maternal deposition of higher levels of T into the last-laid eggs may mitigate the negative effects of hatching asynchrony on chicks hatched from those eggs under good conditions.

In this study, we injected a fixed amount of T and did not account for pre-treatment concentrations of T in the egg yolks. It could then be argued that the dosage we used may have been beyond the physiological range in our study population. However, Tschirren and colleagues [45], in a neighboring population used a fixed amount of T that was twice as high as ours (30 ng vs. 15 ng) and still found positive effects on chicks hatched from last-laid eggs. Moreover, we found a maximum amount of T per yolk of 21 ng in our population (see results and Figure 1). Therefore, adding 15 ng would increase the amount of T up to 36 ng in those eggs and this is close to the maximum concentration found for this species in Switzerland (30 ng/yolk). Although one can argue that this is still above the maximum reported value, it can hardly be considered as supraphysiological with regard to the ca. 60****ng “produced” by Tschirren at al. [45], which yielded positive effects of T. Moreover, 92.5% of the eggs analyzed in our study contained less than 15 ng of T per yolk, meaning that 92.5% of the injected eggs would fall below the highest natural yolk T concentration. We thus believe that, although the fixed dose of T used in our study may have been slightly above the physiological norm for 7.5% of the eggs (at a 30 ng threshold), it was not so for the remaining 92.5%, and thus the detrimental effects found call for another explanation than supraphysiological dosage.

The reduced hatching success in our study led to smaller brood sizes and therefore lowered chick competition. Reduced brood size is well known to improve rearing conditions, and lead to higher fledgling body mass, size, survival, and recruitment rate [68], [79], [80]. Therefore, the results of our experiment will be discussed in a context of good rearing conditions. Reduced hatchability of eggs seems to be a common problem with such injection experiments [81]–[83] and one can argue that only the strongest embryos survived. However, even though the injection may lead to differential survival of embryos, it would affect embryos equally over the treatments since all eggs were injected, and thus have little effects on our results.

In spite of the good conditions generated by the reduced hatching success, we found that first-hatched chicks from first-laid eggs injected with T had similar body mass and wing length as all control chicks from either control broods or T-Last broods (Figures 2 and 4; all post-hoc comparisons P>0.05, see results). First-hatched chicks from first-laid eggs injected with T had tarsi of similar length as their siblings and control chicks from T-Last broods (Figure 3). Their tarsus was longer than for chicks in control broods, but this is due to control-brood chicks being smaller than all chicks from T-injected broods (T-First and T-Last), a result we discuss later. Thus, we overall found no effect on first-hatched chicks of increased T-levels in the first-laid eggs.

Last-hatched chicks from oil-injected eggs in T-First broods achieved similar mass and tarsus length as their T-injected, first-hatched siblings. However, they grew significantly smaller wings than their siblings (see results and Figure 4). This suggests that last-hatched chicks indirectly suffered from elevated yolk T levels in their siblings’ eggs. Females usually deposit more T in the yolk of the last-laid eggs (Figure 1). However, variation exists and this pattern is reversed in some clutches. This last result combined with an absence of positive effect on first-hatched chicks from T-injected eggs renders the adaptive significance of such a reversed pattern elusive. Testing the effects of elevated yolk T in first- vs. last-laid eggs under both good and bad rearing conditions may provide some answers to this question.

The benefit of increasing the concentration of yolk T over the laying sequence is supposedly at least partially counteracting the disadvantage in sibling competition of the last-hatched chicks by enhancing their overall competitiveness under good conditions [31]. In canaries for example, junior chicks that were placed at a competitive disadvantage, similar to later-hatching chicks in natural broods, benefited from elevated yolk T levels via enhanced growth [64]. The same pattern was observed in black-headed gull, where the last-hatched chicks from eggs with elevated levels of T grew heavier and had longer tarsi than the last-hatched chicks from control eggs [62]. In contrast to these previous studies, we found that last-hatched chicks from last-laid eggs injected with T grew smaller and lighter than their oil-injected siblings, and thus seemed to suffer from increased yolk T levels. In these broods, elevated T in last-laid eggs may have induced higher behavioural activity and aggression [84], [85] resulting in higher energy expenditure due to unnecessary increase in metabolism [86], with maladaptive consequences in the current good rearing conditions where sibling competition was lowered. Our results raise the question of the adaptive function of depositing higher amounts of T in the last-laid eggs, as illustrated by the general pattern found here (Figure 1) and by Tschirren et al. [67]. We suggest that high levels of T may be detrimental for the last-laid, last-hatched chicks in general, and particularly under worse rearing conditions. Females would therefore deposit more T in the last-laid eggs to induce brood reduction. This hypothesis could be experimentally tested using the same injection procedure as ours but manipulating post-hatching conditions both to the worse and to the better. A complementary experiment would manipulate both pre-laying and post-hatching conditions to investigate whether females anticipate rearing conditions by varying the amounts of T in the last-laid eggs, and whether patterns of hormone allocation within the laying sequence match a brood reduction strategy.

Our results contradict a study by Tschirren et al. [45] in that we found no positive effect of high yolk T levels on the last-hatched chicks. It is to be noted however that the study by Tschirren and colleagues used a different experimental design. First, by injecting whole clutches either with T or with sesame oil and later using a partial cross-fostering procedure, the authors created mixed broods in which half of the chicks hatched from T-injected eggs and half of the nestlings hatched from oil-injected eggs. Although this design allows testing for an interaction between treatments and laying position, it does not allow for within-clutch comparisons, and is in contrast with our design that aimed to test whether increasing or decreasing amounts of T within the laying sequence is relevant for brood reduction. Also the cross-fostering by itself can induce an additional stress [87]. The discrepancy between our and Tschirren et al.’s method [45] is a strong basis to explain the discrepancy in our respective results. One explanation is that the interposition of control chicks among T chicks in Tschirren et al.’s protocol [45] may have strongly influenced the whole brood dynamics both in terms of sibling rivalry and sibling competition and in terms of parental response to altered begging levels, to such an extent that it interacted with the effect of T on the last-hatched chicks. In our protocol, first- and last-hatched chicks were contrasted in terms of levels of T in the yolk in a manner that should be closer to the natural pattern.

As an alternative explanation of our negative effects on last-hatched chicks it could be postulated that several other substances (e.g. lipids, protein or water) that are found in egg yolk may interact with testosterone, and T concentrations within-clutches may be adjusted to some other components [58]. For instance, differences in lysozyme [88], carotenoid [58] or immunoglobulin content [89] can result in differences in chick growth and fitness. Thus, modifying the concentration of one substance independently of all others may disrupt a subtle balance and thereby lead to detrimental effects on chick growth. Moreover, avian eggs contain also high concentrations of other maternally originated hormones such as dihydrotestosterone (DHT), androstenedione (A4), 17β-oestradiol (E2) and corticosterone (B). Therefore, the injection of only T in our study may modify possible hormonal interactions contributing to unexpected phenotypic changes [9], [90]. On the other hand, it is possible that the eggs may have been prepared for a poor situation in our study and so did not match the good condition created afterwards.

Looking at the average body mass and size of chicks, we found that chicks from control broods had shorter tarsi than chicks from T-First broods (Table 1; mean difference ±1SE: 1.67±0.64 mm, post hoc t-test: t_1,45_ = 2.62, P = 0.012), as well as had shorter tarsi, although non significantly, than chicks from T-Last broods (Table 1; mean difference ±1SE: 1.19±0.63 mm, post hoc t-test: t_1,45_ = 1.88, P = 0.067). One explanation would be that increased levels of T in some eggs of a clutch may change the overall dynamics of the brood, and bring some benefits to the brood as a whole. This idea however is speculative and requires additional examination.

Overall, our results show that females could potentially adjust offspring phenotype by modulating, over the laying sequence, the amounts of T deposited in the eggs. However, they seem to contradict previous findings, and do not support the idea that under good conditions higher levels of maternally derived T in the last-laid eggs may at least partially compensate the negative effects of hatching asynchrony by increasing the last-hatched chicks’ competitive ability.
